# Sensitivity of prehospital stroke scales for different intracranial large vessel occlusion locations

**DOI:** 10.1177/23969873211015861

**Published:** 2021-05-13

**Authors:** Martijne HC Duvekot, Esmee Venema, Hester F Lingsma, Jonathan M Coutinho, H Bart van der Worp, Jeannette Hofmeijer, Reinoud PH Bokkers, Adriaan CGM van Es, Aad van der Lugt, Henk Kerkhoff, Diederik WJ Dippel, Bob Roozenbeek, on behalf of the MR CLEAN Registry investigators

**Affiliations:** 1 Department of Neurology, Albert Schweitzer Hospital, Dordrecht, the Netherlands; 2 Department of Neurology, Erasmus MC University Medical Center, Rotterdam, the Netherlands; 3 Department of Public Health, Erasmus MC University Medical Center, Rotterdam, the Netherlands; 4 Department of Neurology, Amsterdam University Medical Centers, Amsterdam, the Netherlands; 5 Department of Neurology and Neurosurgery, University Medical Center Utrecht, Brain Center, Utrecht, the Netherlands; 6 Department of Neurology, Rijnstate Hospital, Arnhem, the Netherlands; 7 Department of Radiology, Medical Imaging Center, University Medical Center Groningen, University of Groningen, Groningen, the Netherlands; 8 Department of Radiology, Leiden University Medical Center, Leiden, the Netherlands; 9 Department of Radiology and Nuclear Medicine, Erasmus MC University Medical Center, Rotterdam, the Netherlands

**Keywords:** Stroke, endovascular thrombectomy, prehospital stroke scales

## Abstract

**Introduction:**

Prehospital stroke scales have been proposed to identify stroke patients with a large vessel occlusion to allow direct transport to an intervention centre capable of endovascular treatment (EVT). It is unclear whether these scales are able to detect not only proximal, but also more distal treatable occlusions. Our aim was to assess the sensitivity of prehospital stroke scales for different EVT-eligible occlusion locations in the anterior circulation.

**Patients and methods:**

The MR CLEAN Registry is a prospective, observational study in all centres that perform EVT in the Netherlands. We included adult patients with an anterior circulation stroke treated between March 2014 and November 2017. We used National Institutes of Health Stroke Scale scores at admission to reconstruct previously published prehospital stroke scales. We compared the sensitivity of each scale for different occlusion locations. Occlusions were assessed with CT angiography by an imaging core laboratory blinded to clinical findings.

**Results:**

We included 3021 patients for the analysis of 14 scales. All scales had the highest sensitivity to detect internal carotid artery terminus occlusions (ranging from 0.21 to 0.97) and lowest for occlusions of the M2 segment (0.08 to 0.84, p-values < 0.001).

**Discussion and conclusion:** Although prehospital stroke scales are generally sensitive for proximal large vessel occlusions, they are less sensitive to detect more distal occlusions.

## Introduction

Because the effect of endovascular treatment (EVT) for ischaemic stroke is strongly time-dependent, it is important to optimise prehospital and in-hospital workflows to reduce unnecessary treatment delays.^1–3^ Interhospital transfers are an important cause of treatment delay and are associated with worse functional outcome.^4,5^ Prehospital stroke scales may be helpful for the selection of patients with a high likelihood of a large vessel occlusion (LVO), to bypass the primary stroke centre for direct transport to an intervention centre capable of EVT and thereby avoiding time-consuming interhospital transfers.

Numerous prehospital stroke scales have been published over the past few years.^6–20^ These scales have been developed as short and simple clinical tools to identify stroke patients with an LVO. Most scales are derived from the National Institutes of Health Stroke Scale (NIHSS).^21^ Patients with a proximal occlusion usually present with high NIHSS scores, but more distal occlusion locations may be associated with lower NIHSS scores.^22,23^ The sensitivity of prehospital stroke scales in detecting different occlusion locations in LVO is unknown. Because all patients treated with EVT in the Netherlands are registered, we had the opportunity to explore this in a large dataset of patients treated with EVT. We aimed to assess and compare the sensitivity of prehospital stroke scales for the detection of occlusions in different locations in the anterior circulation in a representative cohort of EVT-eligible patients.

## Methods

### Study design

The MR CLEAN (Multicenter Randomized Clinical Trial of Endovascular Treatment for Acute Ischemic Stroke in the Netherlands) Registry is a national, prospective, open, multicentre, observational monitoring study for intervention centres that perform EVT in the Netherlands. We collected data from consecutive patients who underwent EVT in 18 hospitals. Details of the MR CLEAN Registry have been reported previously.^24^

### Prehospital stroke scales

We selected prehospital stroke scales from the literature and included scales that were developed to detect LVO in the anterior circulation. Scales were only included if a cut point was proposed in the original studies. Scales that could not be reproduced with NIHSS items or scales that contained unavailable variables were excluded.

### Study population

All patients with acute ischaemic stroke caused by an intracranial LVO, confirmed by CT angiography (CTA), who had at least a groin puncture as start of EVT, were registered in the MR CLEAN Registry. EVT was performed in all patients with an occlusion of the distal part of the ICA, the M1 or M2 segment of the middle cerebral artery, if treatment was possible within six hours after symptom onset, irrespective of the stroke severity. The only contra-indication was intracranial haemorrhage. Ischaemic stroke in the affected vascular territory in the six weeks prior to the current event was a relative contra-indication. For the purpose of our analysis, we included patients registered between March 16, 2014 and November 1, 2017. We used the following inclusion criteria: age ≥18 years, EVT performed in a centre that participated in the MR CLEAN trial, start of EVT within 6.5 hours after stroke onset, and a proximal intracranial occlusion in the anterior circulation (internal carotid artery (ICA), internal carotid artery terminus (ICA-T), middle cerebral artery (M1/M2)).^24^ We excluded patients of whom CTA was not available. Standard stroke work-up after arrival in the hospital was rapid assessment of the patient, followed by non-contrast CT and CTA. If indicated, intravenous thrombolysis was initiated just prior or after the CTA. Patients who did not present primarily in an intervention centre were transferred for EVT. After transfer and prior to EVT, the NIHSS was assessed by a neurologist or neurology resident in the intervention centre.

### Imaging assessments

All imaging was adjudicated by an imaging core laboratory, whose members were informed about the side of the affected hemisphere. M1 occlusions located before or during the branching off of lenticulostriate arteries were defined as proximal M1 occlusions. M1 occlusions located after the branching off of lenticulostriate arteries were defined as distal M1 occlusions. The M2 segments were defined as the first post-bifurcation branches of the M1 segment. In case of multiple occlusions, the most proximal occlusion location was used for the analysis.

### Statistical analysis

Prehospital stroke scales were reconstructed with the NIHSS items assessed at baseline in the intervention centre. The scales were assessed as positive or negative, using the cut point proposed in the original publication. We calculated the sensitivity for the detection of LVO for each prehospital stroke scale, both stratified by occlusion location and for all occlusion locations combined. For each prehospital stroke scale, the sensitivities for different occlusion locations were compared using Chi-square tests. Additionally, we plotted the sensitivity for all possible cut points of the prehospital stroke scales, stratified by occlusion location. Potential differences in sensitivity across prehospital stroke scales may be caused by variation in the included NIHSS items. Therefore, we calculated the percentage of patients in our cohort who had an abnormal score on each NIHSS item. All analyses were performed using R software version 3.6.1 and Rstudio version 1.0.153.

## Results

**Figure 1.**
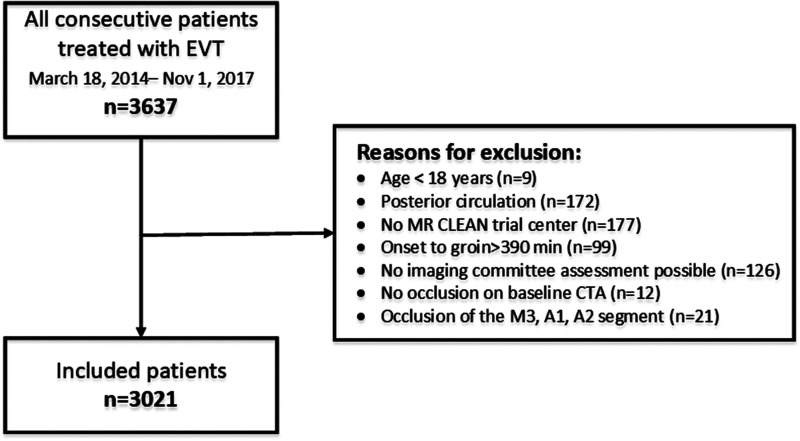
Selection of study population.

Fourteen prehospital stroke scales were available for our analysis (Table S1 supplemental material).^6–20^ In total, 3637 patients were registered in the MR CLEAN Registry between March 16, 2014 and November 1, 2017. We excluded 616 patients who did not meet our inclusion criteria (Figure 1). Of the 3021 included patients, the median age was 72 years and 52% of the patients were men (Table 1). Most patients, 1333 of 3021 (44%) had a baseline NIHSS of 17 or higher, but 190 patients (6%) had a low baseline NIHSS, ranging from zero to four. The most common occlusion location was the distal M1 segment (n = 1026, 34%). The least common occlusion locations were the M2 segment (n = 462, 15%) and the intracranial ICA (n = 155, 5%).

**Table 1.** Baseline characteristics of the 3021 included patients.

Characteristics	*N* = 3021	Missings
Age, median (IQR)	72 (61–81)	0
Male sex	1564 (52%)	0
Occlusion side: left hemisphere	1601 (53%)	0
Baseline NIHSS		0
0–4	190 (6%)	
5–8	323 (11%)	
9–12	466 (15%)	
13–16	709 (24%)	
≥17	1333 (44%)	
Systolic blood pressure, mean ± SD	150 ± 25	83 (2.7%)
Treatment with IVT	2309 (76%)	7 (0.2%)
Medical history		
Previous stroke	501 (17%)	27 (0.9%)
Atrial fibrillation	727 (24%)	40 (1.3%)
Diabetes mellitus	475 (16%)	23 (0.08%)
Myocardial infarction	416 (14%)	59 (2.0%)
Hypertension	1545 (51%)	66 (2.2%)
Pre-stroke mRS		65 (2.2%)
0–2	2612 (86%)	–
≥3	344 (11%)	–
Transferred to intervention centre	1650 (55%)	1 (0.03%)
Onset-to-door time in minutes, median (IQR)	132 (62–188)	146 (4.8%)
Door-to-CTA-time in minutes^a^, median (IQR)	15 (−64–27)	732 (24.2%)
Door-to-needle-time in minutes, median (IQR)	24 (18–33)	495 (16.4%)
Door-to-groin-time in minutes^a^, median (IQR)	60 (35–90)	267 (8.8%)
ASPECTS at baseline, median (IQR)	9 (8–10)	61 (2.2%)
Collateral score at baseline		86 (2.8%)
Grade 0	185 (6%)	–
Grade 1	1063 (35%)	–
Grade 2	1143 (38%)	–
Grade 3	544 (18%)	–
Level of occlusion on CTA^b^		0
Intracranial ICA	155 (5%)	–
ICA-T	640 (21%)	–
Proximal M1	738 (24%)	–
Distal M1	1026 (34%)	–
M2	462 (15%)	–

IQR: interquartile range; SD: standard deviation; NIHSS: National Institutes of Health Stroke Scale; IVT: intravenous thrombolysis; mRS: modified Rankin Scale; ASPECTS: Alberta Stroke Program Early CT Score; ICA: internal carotid artery.

Values are expressed in numbers (%) unless otherwise indicated.

^a^Door-to-CTA-time and door-to-groin-time were calculated using the door-time of the intervention centre.

^b^Percentages do not add up to 100% due to rounding.

**Figure 2.**
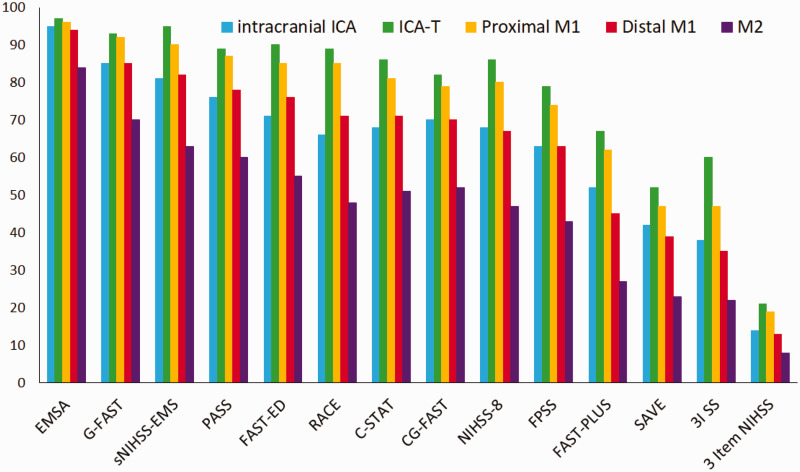
Bar plots of the sensitivity per stroke scale, stratified by occlusion location.

For all scales, sensitivity was highest for ICA-T occlusions, with sensitivities ranging from 0.21 to 0.97 (Table 2). Sensitivities decreased for the more distal occlusion segments as well as for the intracranial ICA compared to ICA-T occlusions. M2 occlusions were least likely to be detected, with sensitivities ranging from 0.08 to 0.84 (Figure 2). The difference in sensitivity between occlusion locations was significant for all scales (p < 0.001). The Emergency Medical Stroke Assessment (EMSA) and Gaze-Face-Arm-Speech-Time (G-FAST) had the highest sensitivity for all different occlusion locations. The Speech Arm Vision Eyes Scale (SAVE), 3-Item Stroke Scale (3I SS), and three-item NIHSS had the lowest sensitivity, for all different occlusion locations. The sensitivity of prehospital stroke scales to detect LVO for all occlusion locations together also varied widely, from 0.15 to 0.94. Sensitivity of the prehospital stroke scales for all possible cut points, stratified by occlusion location, are provided in Figures S1 to S5 of the supplement.

**Table 2.** Sensitivity (with 95% confidence interval) for the detection of LVO by 14 prehospital stroke scales in the full study cohort and stratified by occlusion location.

Prehospital stroke scale	Full cohort	Intracranial ICA	ICA-T	Proximal M1	Distal M1	M2	*p* value
EMSA ≥ 3	0.94 (0.93–0.94)	0.95 (0.91–0.98)	0.97 (0.96–0.99)	0.96 (0.95–0.97)	0.94 (0.92–0.95)	0.84 (0.80–0.87)	<0.001
G-FAST ≥ 3	0.86 (0.85–0.87)	0.85 (0.79–0.90)	0.93 (0.92–0.95)	0.92 (0.89–0.94)	0.85 (0.82–0.87)	0.70 (0.65–0.73)	<0.001
sNIHSS-EMS ≥ 6	0.84 (0.68–0.72)	0.81 (0.75–0.87)	0.95 (0.93–0.97)	0.90 (0.88–0.93)	0.82 (0.80–0.85)	0.63 (0.59–0.68)	<0.001
PASS ≥ 2	0.79 (0.78–0.81)	0.76 (0.69–0.82)	0.89 (0.87–0.91)	0.87 (0.84–0.89)	0.78 (0.75–0.80)	0.60 (0.55–0.64)	<0.001
FAST-ED ≥ 4	0.78 (0.76–0.79)	0.71 (0.64–0.78)	0.90 (0.88–0.92)	0.85 (0.82–0.87)	0.76 (0.73–0.78)	0.55 (0.50–0.60)	<0.001
RACE ≥5	0.75 (0.73–0.76)	0.66 (0.58–0.73)	0.89 (0.87–0.92)	0.85 (0.83–0.88)	0.71 (0.68–0.74)	0.48 (0.43–0.52)	<0.001
C-STAT ≥ 2	0.73 (0.72–0.75)	0.68 (0.60–0.75)	0.86 (0.83–0.89)	0.81 (0.78–0.84)	0.71 (0.68–0.73)	0.51 (0.46–0.55)	<0.001
CG-FAST ≥ 4	0.72 (0.71–0.74)	0.70 (0.62–0.77)	0.82 (0.79–0.85)	0.79 (0.76–0.82)	0.70 (0.68–0.73)	0.52 (0.47–0.57)	<0.001
NIHSS-8 ≥ 8	0.71 (0.69–0.73)	0.68 (0.61–0.76)	0.86 (0.86–0.89)	0.80 (0.77–0.83)	0.67 (0.64–0.70)	0.47 (0.42–0.51)	<0.001
FPSS ≥ 5	0.66 (0.64–0.68)	0.63 (0.55–0.70)	0.79 (0.75–0.82)	0.74 (0.71–0.77)	0.63 (0.60–0.66)	0.43 (0.38–0.47)	<0.001
FAST-PLUS positive^a^	0.52 (0.50–0.53)	0.52 (0.44–0.60)	0.67 (0.64–0.71)	0.62 (0.58–0.65)	0.45 (0.42–0.48)	0.27 (0.23–0.31)	<0.001
SAVE ≥ 4	0.42 (0.40–0.43)	0.42 (0.34–0.50)	0.52 (0.48–0.56)	0.47 (0.44–0.51)	0.39 (0.36–0.42)	0.23 (0.18–0.26)	<0.001
3I SS ≥ 4	0.42 (0.39–0.43)	0.38 (0.30–0.46)	0.60 (0.58–0.63)	0.47 (0.43–0.50)	0.35 (0.32–0.38)	0.22 (0.19–0.26)	<0.001
3 Item NIHSS ≥ 5	0.15 (0.14–0.17)	0.14 (0.08–0.19)	0.21 (0.18–0.24)	0.19 (0.17–0.22)	0.13 (0.11–0.15)	0.08 (0.05–0.10)	<0.001

ICA: internal carotid artery; EMSA: Emergency Medical Stroke Assessment; G-FAST: Gaze-Face-Arm-Speech-Time; sNIHSS-EMS: shortened NIH Stroke Scale for emergency medical services; PASS: Prehospital Acute Stroke Severity scale; FAST-ED: Field Assessment Stroke Triage for Emergency Destination; RACE: Rapid Arterial oCclusion Evaluation; C-STAT: Cincinnati Stroke Triage Assessment Tool; CG-FAST: Conveniently-Grasped Field Assessment Stroke Triage; NIHSS-8: National Institutes of Health Stroke Scale-8; FPSS: Finnish Prehospital Stroke Scale; SAVE: Speech Arm Vision Eyes Scale; 3I SS: 3-Item Stroke Scale.

^a^FAST-PLUS is an algorithm and does not have a cut point.

The NIHSS items motor arm, aphasia and dysarthria (combined in one item), and facial paresis were the most frequently affected items in our cohort (Table 3). The scales with the highest sensitivity mainly consisted of commonly affected items, whereas the scales with the lowest sensitivity consisted largely of the least affected items.

**Table 3.** Overview of prehospital stroke scales.

Prehospital stroke scales	Cut point/total score	Motor arm	Language and dysartria^a^	Facial paresis	Motor leg	Dysartria	Gaze	LOC questions	Aphasia	Visual fields	Sensation	Extinction	LOC commands	LOC responsiveness
Abnormal score per item	–	91%	90%	87%	84%	68%	66%	59%	57%	55%	49%	49%	42%	21%
EMSA	3/6	●	●	●	●		●							
G-FAST	3/4	●	●	●			●							
sNIHSS-EMS	6/29	●		●	●	●			●		●			●
PASS	2/3	●					●	●						
FAST-ED	4/9	●	●	●			●					●		
RACE	5/9	●		●	●		●					●	●	
C-STAT	2/4	●					●	●					●	
CG-FAST	4/5	●	●	●			●	●						
NIHSS-8	8/24	●		●		●	●	●				●	●	●
FPSS	5/8	●	●	●	●		●			●				
FAST-PLUS	^b^	●	●	●	●									
SAVE	4/4	●	●				●			●				
3I SS	4/6	●			●		●							●
3 Item NIHSS	5/8	●									●	●		

EMSA: Emergency Medical Stroke Assessment; GFAST: Gaze-Face-Arm-Speech-Time; sNIHSS-EMS: shortened NIH Stroke Scale for emergency medical services; PASS: Prehospital Acute Stroke Severity scale; FAST-ED: Field Assessment Stroke Triage for Emergency Destination; RACE: Rapid Arterial oCclusion Evaluation; C-STAT: Cincinnati Stroke Triage Assessment Tool; CG-FAST: Conveniently-Grasped Field Assessment Stroke Triage; NIHSS-8: National Institutes of Health Stroke Scale-8; FPSS: Finnish Prehospital Stroke Scale; SAVE: Speech Arm Vision Eyes Scale; 3I SS: 3-Item Stroke Scale.

Prehospital stroke scales are ordered based on their sensitivity in the full cohort, from high to low. Percentage of the abnormal score per item is the percentage of patients with a score > 0 for the corresponding NIHSS item. NIHSS items are ordered based on this percentage, from high to low.

^a^In most prehospital stroke scales, NIHSS item 9 (language) and 10 (speech) were merged.

^b^FAST-PLUS is an algorithm and does not have a cut point.

## Discussion

In this study, we demonstrated large differences in sensitivity of the prehospital stroke scales between different occlusion locations. In general, prehospital stroke scales are most sensitive to detect ICA-T occlusions and least sensitive to detect M2 occlusions.

The decrease in sensitivity of prehospital stroke scales for more distal occlusion locations can be explained by the cerebrovascular anatomy. Proximal occlusions affect a larger brain territory than distal occlusions, which generally results in more severe clinical symptoms. This general rule does not apply to the intracranial ICA, probably due to the collateral function of the circle of Willis.

The variation in sensitivity between different scales can be largely explained by the cut point that is used and the likelihood of its scale-items being affected. For example, the most sensitive scale, EMSA, has a low cut point of three out of six, containing the four most frequently affected items. The least sensitive scales, 3I SS and the Three Item NIHSS were both constructed out of less frequently affected items, and they have relatively high cut points, which resulted in low sensitivity. In addition, some scales (e.g. 3I SS, Rapid Arterial oCclusion Evaluation (RACE), G-FAST, Cincinnati Stroke Triage Assessment Tool (C-STAT), and NIHSS-8) were not primarily designed to detect isolated M2 occlusions.

So far, no studies have focused on the sensitivity of prehospital stroke scales for different occlusion locations. Only one study briefly addressed the sensitivity of the FPSS per occlusion location and was in accordance with our findings.^16^ One other study showed that in patients with a Field Assessment Stroke Triage for Emergency Destination (FAST-ED) < 4, a higher prevalence of M2 occlusions was found than in patients with FAST-ED ≥ 4.^13^ A validation study of the RACE scale demonstrated M1 and M2 occlusions will be missed more often than ICA-T occlusions.^25^ Furthermore, in two separate studies, subgroup analyses excluding M2 occlusions showed a higher sensitivity for the RACE scale, 3I SS and C-STAT.^26,27^

The MR CLEAN Registry is a large nationwide registry including all patients treated with EVT. All baseline CTAs were assessed by an experienced imaging core laboratory, providing accurate information about the occlusion location. Previously reported sensitivities of prehospital stroke scales could have been influenced by the distribution of the different occlusion locations within the validated cohort. Since our cohort is an unselected representation of patients treated with EVT, it reflects daily clinical practice. Nevertheless, we did not include undiagnosed LVO patients (because CTA was omitted) or untreated LVO patients. However, we expect that this bias will be limited because the Dutch national guideline recommends CTA in all ischaemic stroke patients.^28^ Furthermore, due to the broad EVT treatment criteria in this guideline, almost all LVO patients are treated. Only sporadically, patients with low NIHSS, mostly in combination with distal occlusions such as the M2 segment, will not be treated. Therefore, the sensitivity to detect occlusions in the M2 segment might be slightly overestimated. However, even if we would have been able to include the small number of untreated LVO patients, we expect the effect on our results to be limited. We cannot fully exclude between-centre differences in EVT indications. We did not account for this in the statistical analysis because potential centre differences might also be explained by differences in case-mix and this falls out of the scope of this study. In our opinion, the multicentre nature of the study is a strength, which allowed us to stratify for occlusion location in a large representative cohort of the Dutch EVT population.

Our study has some limitations. We reconstructed prehospital stroke scale scores based on the NIHSS performed by experienced physicians at the emergency department. Prehospital stroke scales should be validated in a prehospital setting by paramedics, as this is the setting in which the scales will be used. However, a prehospital study that acquires substantial numbers for every occlusion location is practically impossible to carry out. It would require a very large sample size.^29^ Even though scale assessment by paramedics might differ from the assessment of experienced physicians, we expect the overall decay in sensitivity towards more distal occlusion locations will also apply in the prehospital assessments by paramedics. Additionally, there is some evidence that prehospital assessments are comparable with assessments by physicians, as demonstrated for the RACE and FAST-ED.^30,31^ Because we did not include patients with an LVO in the anterior cerebral artery (A1/A2), we were not able to calculate the sensitivity for A1/A2 occlusions. However, isolated A1/A2 occlusions are uncommon and our cohort counted only 12 (0.3%) of those occlusions. Unfortunately, we could not include all published prehospital stroke scales, as some scales could not be derived from NIHSS items. For example, the commonly used Los Angeles Motor Scale (LAMS) contains the item “grip strength”, which is not incorporated in the NIHSS, and the ambulance clinical triage for acute stroke treatment (ACT-FAST) algorithm also contains several items that were unavailable.^7,32^

The design of the MR CLEAN Registry allowed us to assess the sensitivity of prehospital stroke scales for different occlusion locations. However, our study does not provide sufficient information to decide on the most accurate scale, because our cohort only consists of patients with LVO. This does not allow us to calculate other diagnostic test parameters of the prehospital stroke scales, such as specificity. The ideal prehospital stroke scale is based on a trade-off between sensitivity and specificity. Prospective, prehospital validation studies such as the recently published PRESTO study and a similar study provide a better insight in the prehospital stroke scale performance.^33,34^ However, in these studies it was not possible to assess the sensitivity of different occlusion locations because of the relatively small numbers of LVO patients. Finally, since endovascular treatment possibilities are developing further, the added value of prehospital stroke scales to detect LVO patients in the delayed time window or to detect more distal occlusion locations needs to be investigated.

## Conclusions

The sensitivity of prehospital stroke scales varies widely between different occlusion locations. Our study demonstrates that prehospital stroke scales are most sensitive in detecting ICA-T occlusions and least sensitive in detecting M2 occlusions. Since the treatment of isolated M2 occlusions is considered effective and safe,^22,23^ it is important to realise that a considerable proportion of treatable LVO patients will be missed.

## Supplemental Material

sj-pdf-1-eso-10.1177_23969873211015861 - Supplemental material for Sensitivity of prehospital stroke scales for different intracranial large vessel occlusion locations Click here for additional data file. Supplemental material, sj-pdf-1-eso-10.1177_23969873211015861 for Sensitivity of prehospital stroke scales for different intracranial large vessel occlusion locations by Martijne HC Duvekot, Esmee Venema, Hester F Lingsma, Jonathan M Coutinho, H Bart van der Worp, Jeannette Hofmeijer, Reinoud PH Bokkers, Adriaan CGM van Es, Aad van der Lugt, Henk Kerkhoff, Diederik WJ Dippel, Bob Roozenbeek and on behalf of the MR CLEAN Registry investigators in European Stroke Journal

## Research nurses/local trial coordinators

Naziha el Ghannouti1; Martin Sterrenberg1; Wilma Pellikaan7; Rita Sprengers4; Marjan Elfrink11; Michelle Simons11; Marjolein Vossers12; Joke de Meris14; Tamara Vermeulen14; Annet Geerlings19; Gina van Vemde22; Tiny Simons30; Gert Messchendorp^28^; Nynke Nicolaij28; Hester Bongenaar32; Karin Bodde24; Sandra Kleijn34; Jasmijn Lodico34; Hanneke Droste34; Maureen Wollaert5; Sabrina Verheesen5; D Jeurrissen5; Erna Bos9; Yvonne Drabbe15; Michelle Sandiman15; Nicoline Aaldering11; Berber Zweedijk17; Jocova Vervoort21; Eva Ponjee22; Sharon Romviel19; Karin Kanselaar19; Denn Barning10.

## PhD/medical students

Esmee Venema^40^; Vicky Chalos1^,^40; Ralph R Geuskens3; Tim van Straaten19;Saliha Ergezen1; Roger RM Harmsma1; Daan Muijres1; Anouk de Jong1; Olvert A Berkhemer1^,^3^,^6; Anna MM Boers3^,^39; J Huguet3; PFC Groot3; Marieke A Mens3; Katinka R van Kranendonk3; Kilian M Treurniet3; Manon L Tolhuisen3^,^39; Heitor Alves3; Annick J Weterings3; Eleonora LF Kirkels3; Eva JHF Voogd11; Lieve M Schupp^3^; Sabine L Collette28^,^29; Adrien ED Groot4; Natalie E LeCouffe4; Praneeta R Konduri^39^; Haryadi Prasetya^39^; Nerea Arrarte-Terreros^39^; Lucas A Ramos^39^.

## List of affiliations

Department of Neurology1, Radiology2, Public Health^40^, Erasmus MC University Medical Center;

Department of Radiology and Nuclear Medicine3, Neurology4, Biomedical Engineering & Physics^39^, Amsterdam UMC, University of Amsterdam, Amsterdam;

Department of Neurology5, Radiology6, Maastricht University Medical Center and Cardiovascular Research Institute Maastricht (CARIM);

Department of Neurology7, Radiology8, Sint Antonius Hospital, Nieuwegein;

Department of Neurology9, Radiology10, Leiden University Medical Center;

Department of Neurology11, Radiology12, Rijnstate Hospital, Arnhem;

Department of Radiology13, Neurology14, Haaglanden MC, the Hague;

Department of Neurology15, Radiology16, HAGA Hospital, the Hague;

Department of Neurology17, Radiology18, University Medical Center Utrecht;

Department of Neurology19, Neurosurgery20, Radiology27, Radboud University Medical Center, Nijmegen;

Department of Neurology21, Radiology26, Elisabeth-TweeSteden ziekenhuis, Tilburg;

Department of Neurology22, Radiology23, Isala Klinieken, Zwolle;

Department of Neurology24, Radiology25, Reinier de Graaf Gasthuis, Delft;

Department of Neurology28, Radiology29, University Medical Center Groningen;

Department of Neurology30, Radiology31, Atrium Medical Center, Heerlen;

Department of Neurology32, Radiology33, Catharina Hospital, Eindhoven;

Department of Neurology34, Radiology^35^, Medical Spectrum Twente, Enschede;

Department of Radiology^36^, Amsterdam UMC, Vrije Universiteit van Amsterdam, Amsterdam;

Department of Radiology^37^, Noordwest Ziekenhuisgroep, Alkmaar;

Department of Radiology^38^, Texas Stroke Institute, Texas, United States of America.
