# Supplementary material for: Sensitivity of prehospital stroke scales for different intracranial large vessel occlusion locations
Source: Eur Stroke J. 2021 May 13;6(2):194–204. doi: 10.1177/23969873211015861 (PMC8370085; doi:10.1177/23969873211015861)
Supplement: sj-pdf-1-eso-10.1177_23969873211015861 - Supplemental material for Sensitivity of prehospital stroke scales for different intracranial large vessel occlusion locations [file sj-pdf-1-eso-10.1177_23969873211015861.pdf]

Supplementary material

Sensitivity of prehospital stroke scales for different intracranial large vessel occlusion locations

Table 1: Prehospital stroke scales with corresponding NIHSS items and breakdowns

Figure 1: Sensitivity of prehospital stroke scales for intracranial ICA occlusions

Figure 2: Sensitivity of prehospital stroke scales for ICA-T occlusions

Figure 3: Sensitivity of prehospital stroke scales for proximal M1 occlusions

Figure 4: Sensitivity of prehospital stroke scales for distal M1 occlusions

Figure 5: Sensitivity of prehospital stroke scales for M2 occlusions

Table 1. Prehospital stroke with corresponding NIHSS items and breakdowns.

Item and NIHSS correspondence	RACE[1]	C-STAT[2]	PASS[3]	G-FAST[4]	FAST-ED[5]	FAST-PLUS[6]	EMSA[7]	CG-FAST[8]	NIHSS-8[9]	3I SS[10]	SAVE[11]	FPSS[12]	sNIHSS-EMS[13]	3 Item NIHSS[14]
**LOC responsiveness - 1a**														
0 - Alert									0	0			0	
1 - Rousable to minor stimulation									1	1			1	
2 - Rousable to painful stimulation									2	2			2	
3 - Reflex response/unrousable									3				3	
**LOC questions - 1b**														
0 – answers correctly		0	0					0	0					
1 – incorrectly answer one question			1					1	1					
2 – incorrectly answers both questions		1^							2					
**LOC commands - 1c**														
0 – performs both tasks	0*	0							0					
1 - performs one task	1*	1^							1					
2- performs neither tasks	2*								2					
**Best gaze - 2**														
0 - normal	0	0	0	0	0		0	0	0	0	0	0		
1 – partial gaze palsy	1	2	1	1	1		1	1	1	1	1	4		
2 – forced deviation					2				2	2				
**Visual fields - 3**														
No visual loss											0	0		
Partial hemianopia											1	1		
Complete hemianopia														
Bilateral hemianopia														
**Facial paresis - 4**														
0 – normal	0			0	0	0	0	0	0			0	0	
1 – minor paralysis	1			1		1	1	1	1			1	1	
2 – partial paralysis	2				1				2				2	
3 – complete paralysis									3				3	
**Arm - 5a and 5b**									L / R				L / R	
0 – no drift	0	0	0	0	0	0	0	0	0 / 0	0~	0	0~	0 / 0	0
1 – drift			1	1	1	1 / 0#	1		1 / 1	1~	1∞	1~	1 / 1	1
2 – some effort against gravity	1	1						1	2 / 2	2~			2 / 2	2
3 – no effort against gravity	2				2	1#			3 / 3				3 / 3	3
4 – no movement									4 / 4				4 / 4	4
**Leg - 6a and 6b**													L / R	
0 – no drift	0					0#	0			0~		0~	0 / 0	
1 – drift							1**			1~		1~	1 / 1	
2 – some effort against gravity	1									2~			2 / 2	
3 – no effort against gravity	2					1#							3 / 3	
4 – no movement													4 / 4	
**Sensory - 8**														
0 - normal													0	0
1 - mild-moderate loss													1	1
2 - complete loss													2	2

Language & Dysartria - 9 and 10									only 10				9	10	
0 – absent				0	0	0	0	0	0		0	0	0	0	
1 – mild to moderate					1				1				1	1	
2 – severe				1	2	1	2	1	2		1	1	2	2	
3 - mute									x				3	x	
**Neglect - 11**															
0 – absent	0ǂ				0				0						0
1 – extinction in one modality	1ǂ				1				1						1
2 – extinction in more than one modality	2ǂ				2				2						2

^ 1 point if only one of questions and 1 of the commands performed

§ only answers

* if right hemiparesis

ǂ if left hemiparesis

score only if FAST positive

** 1 point if item left ≠ right

~ score highest of four extremities

∞ only scored when asymmetric drift or weakness

RACE: Rapid Arterial oCclusion Evaluation, , C-STAT: Cincinnati Stroke Triage Assessment Tool, PASS: Prehospital Acute Stroke Severity scale, G-FAST: Gaze-Face-Arm-Speech-Time, FAST-ED: Field Assessment Stroke Triage for Emergency Destination, EMSA: Emergency Medical Stroke Assessment, CG-FAST: Conveniently-Grasped Field Assessment Stroke Triage, NIHSS-8: National Institutes of Health Stroke Scale-8, 3I SS: 3-Item Stroke Scale, SAVE: Speech Arm Vision Eyes Scale, FPSS: Finnish Prehospital Stroke Scale, sNIHSS-EMS: shortened NIH Stroke Scale for emergency medical services.

Figure 1: Sensitivity of prehospital stroke scales for intracranial ICA occlusions

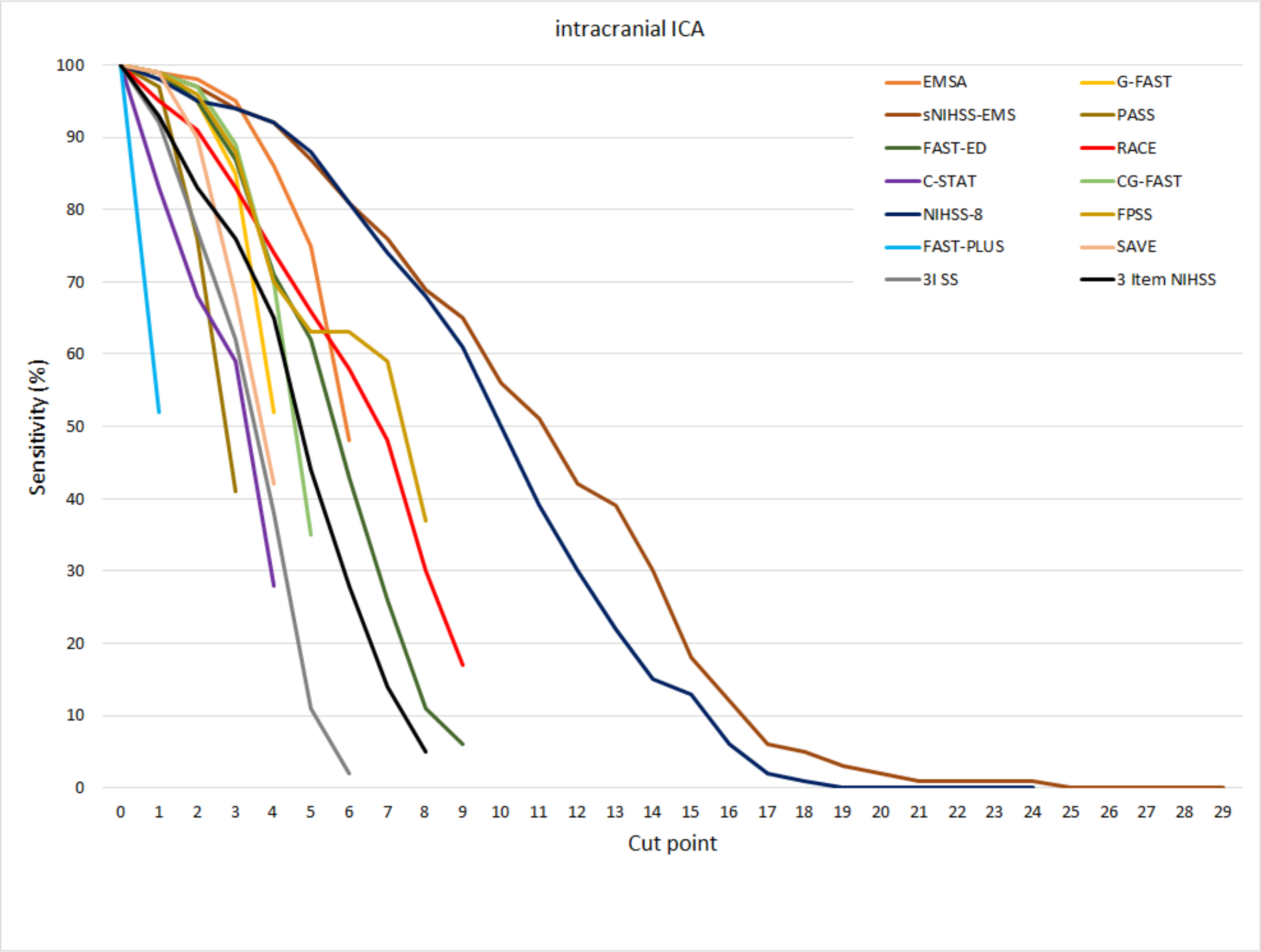

Figure 2: Sensitivity of prehospital stroke scales for ICA-T occlusions

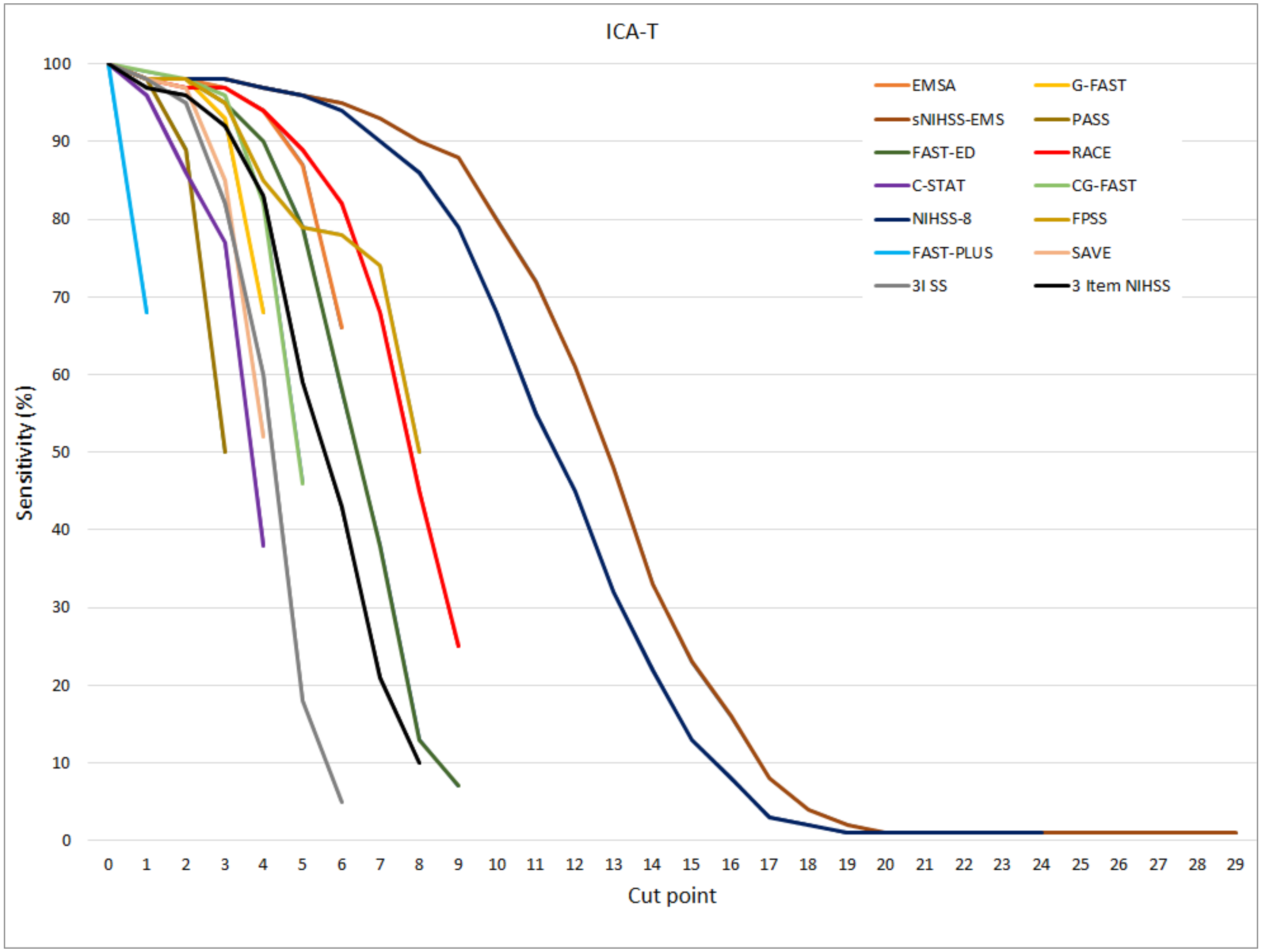

Figure 3: Sensitivity of prehospital stroke scales for proximal M1 occlusions

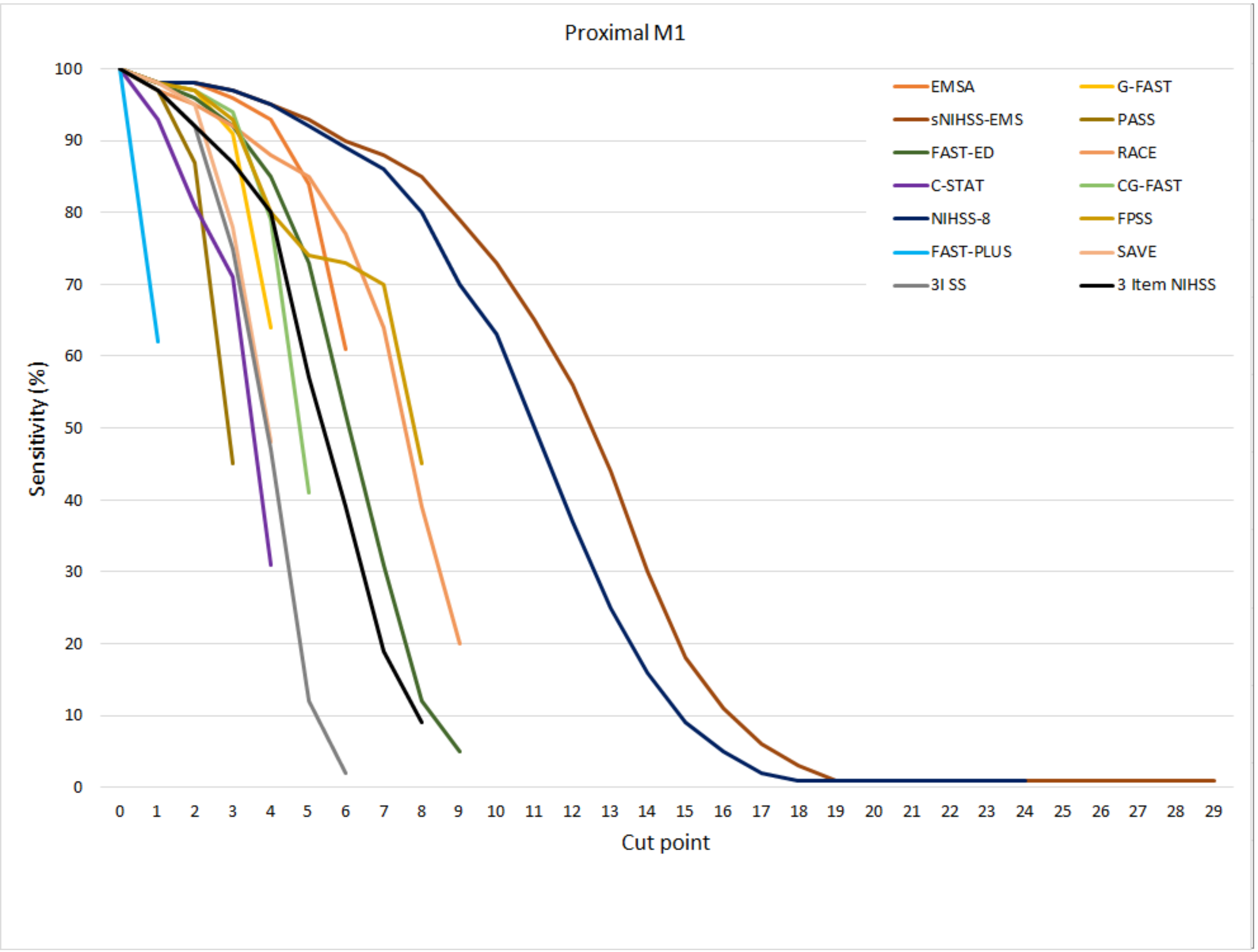

Figure 4: Sensitivity of prehospital stroke scales for distal M1 occlusions

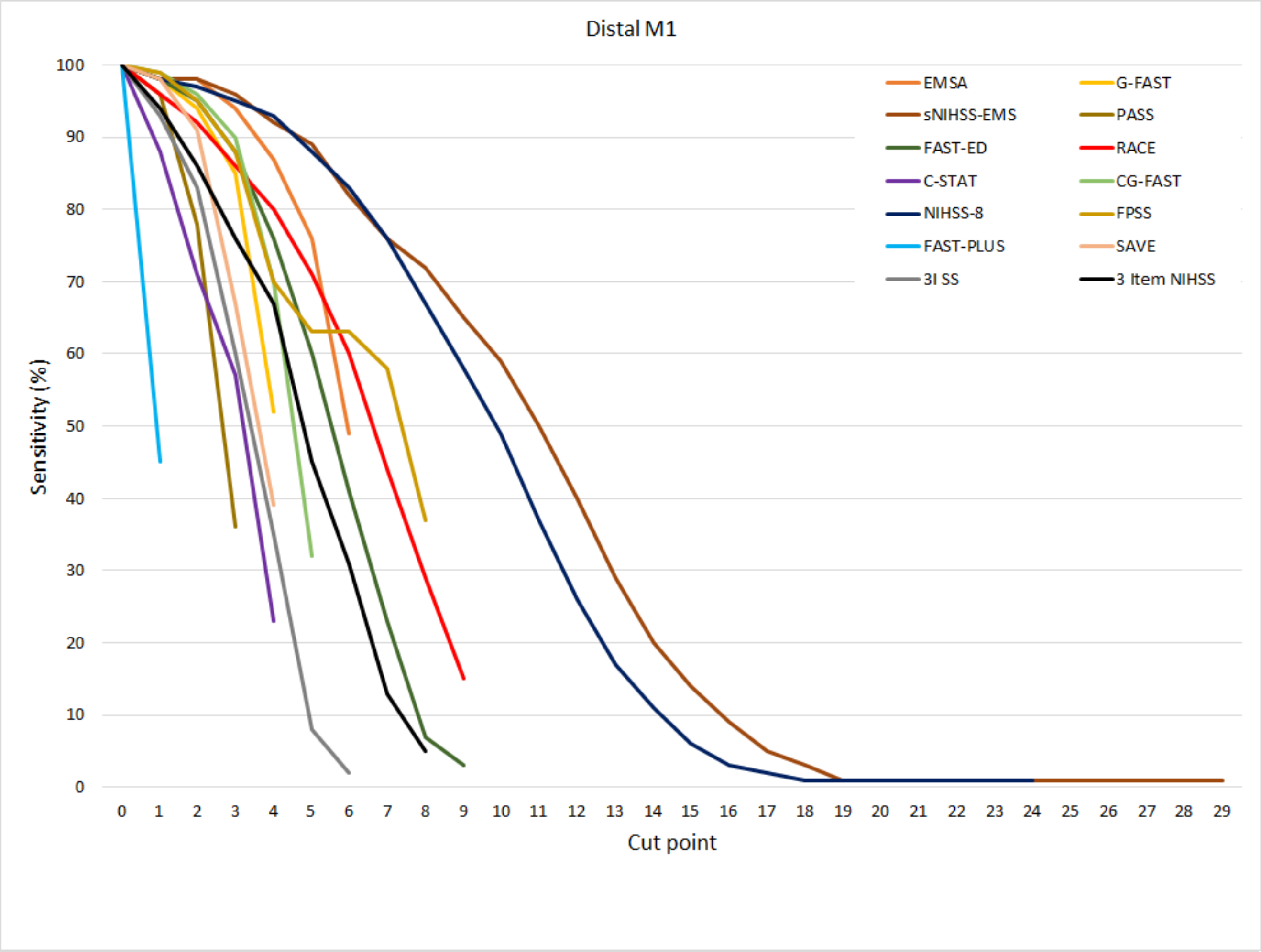

Figure 5: Sensitivity of prehospital stroke scales for M2 occlusions

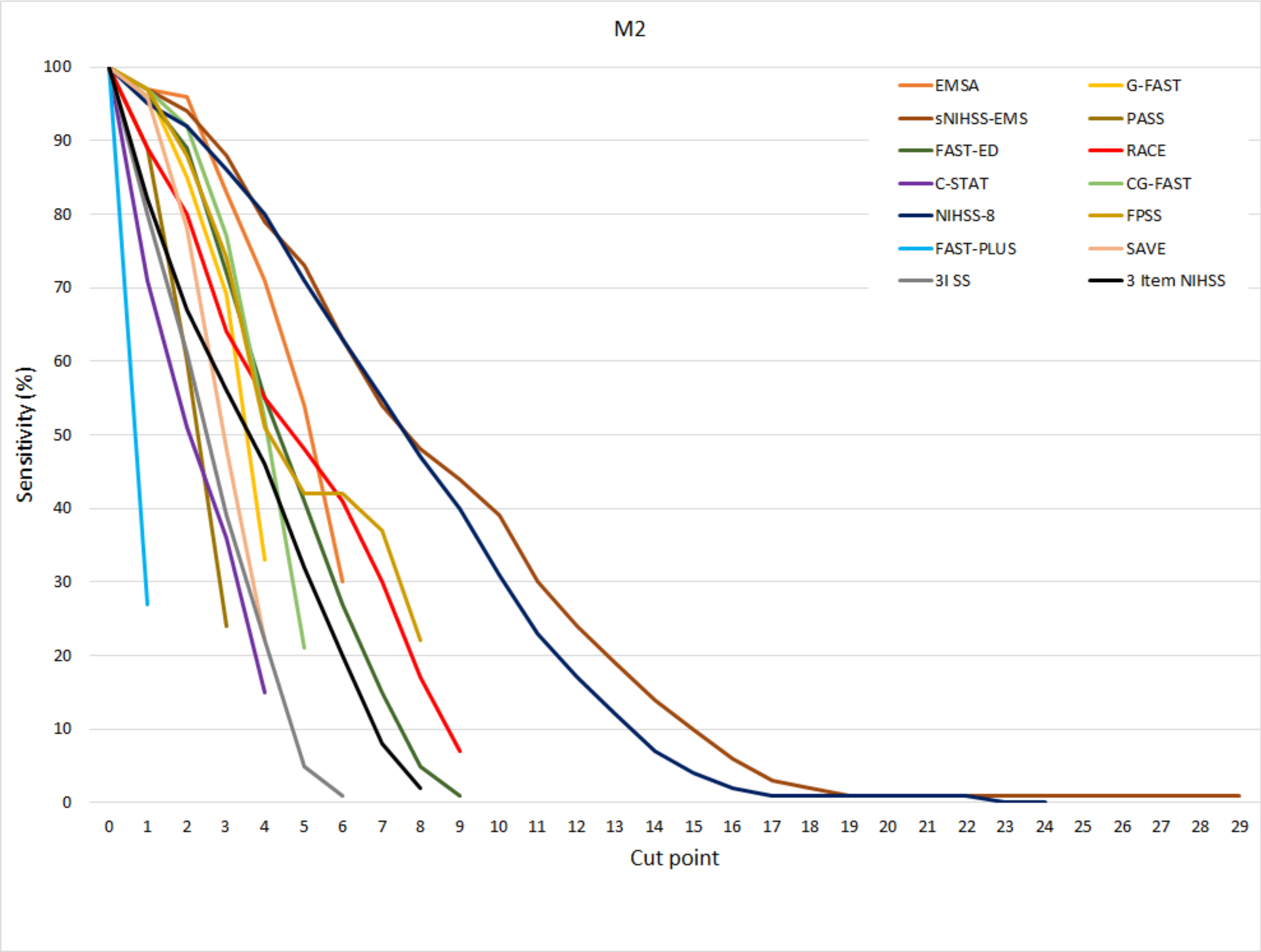

References

1. Perez de la Ossa N, Carrera D, Gorchs M, Querol M, Millan M, Gomis M, et al. Design and validation of a prehospital stroke scale to predict large arterial occlusion: The rapid arterial occlusion evaluation scale. *Stroke*. 2014;45:87-91
2. Katz BS, McMullan JT, Sucharew H, Adeoye O, Broderick JP. Design and validation of a prehospital scale to predict stroke severity: Cincinnati prehospital stroke severity scale. *Stroke*. 2015;46:1508-1512
3. Hastrup S, Damgaard D, Johnsen SP, Andersen G. Prehospital acute stroke severity scale to predict large artery occlusion: Design and comparison with other scales. *Stroke*. 2016;47:1772-1776
4. Scheitz JF, Abdul-Rahim AH, MacIsaac RL, Cooray C, Sucharew H, Kleindorfer D, et al. Clinical selection strategies to identify ischemic stroke patients with large anterior vessel occlusion: Results from sits-istr (safe implementation of thrombolysis in stroke international stroke thrombolysis registry). *Stroke*. 2017;48:290-297
5. Lima FO, Silva GS, Furie KL, Frankel MR, Lev MH, Camargo EC, et al. Field assessment stroke triage for emergency destination: A simple and accurate prehospital scale to detect large vessel occlusion strokes. *Stroke*. 2016;47:1997-2002
6. Vaclavik D, Bar M, Klecka L, Holes D, Cabal M, Mikulik R. Prehospital stroke scale (fast plus test) predicts patients with intracranial large vessel occlusion. *Brain Behav*. 2018:e01087
7. Gropen TI, Boehme A, Martin-Schild S, Albright K, Samai A, Pishanidar S, et al. Derivation and validation of the emergency medical stroke assessment and comparison of large vessel occlusion scales. *J Stroke Cerebrovasc Dis*. 2018;27:806-815
8. Gong X, Chen Z, Shi F, Zhang M, Xu C, Zhang R, et al. Conveniently-grasped field assessment stroke triage (cg-fast): A modified scale to detect large vessel occlusion stroke. *Front Neurol*. 2019;10:390
9. Demeestere J, Garcia-Esperon C, Lin L, Bivard A, Ang T, Smoll NR, et al. Validation of the national institutes of health stroke scale-8 to detect large vessel occlusion in ischemic stroke. *J Stroke Cerebrovasc Dis*. 2017;26:1419-1426
10. Singer OC, Dvorak F, du Mesnil de Rochemont R, Lanfermann H, Sitzer M, Neumann-Haefelin T. A simple 3-item stroke scale: Comparison with the national institutes of health stroke scale and prediction of middle cerebral artery occlusion. *Stroke*. 2005;36:773-776
11. Keenan KJ, Smith WS. The speech arm vision eyes (save) scale predicts large vessel occlusion stroke as well as more complicated scales. *J Neurointerv Surg*. 2018
12. Ollikainen JP, Janhunen HV, Tynkkynen JA, Mattila KM, Halinen MM, Oksala NK, et al. The finnish prehospital stroke scale detects thrombectomy and thrombolysis candidates-a propensity score-matched study. *J Stroke Cerebrovasc Dis*. 2018;27:771-777
13. Purrucker JC, Hartig F, Richter H, Engelbrecht A, Hartmann J, Auer J, et al. Design and validation of a clinical scale for prehospital stroke recognition, severity grading and prediction of large vessel occlusion: The shortened nih stroke scale for emergency medical services. *BMJ Open*. 2017;7:e016893
14. Zuckerman SL, Sivaganesan A, Zhang C, Dewan MC, Morone PJ, Ganesh Kumar N, et al. Maximizing efficiency and diagnostic accuracy triage of acute stroke patients: A case-control study. *Interv Neuroradiol*. 2016;22:304-309
